# Visualization of oxygen profile in reconstructed human epidermis by phosphorescence-lifetime imaging microscopy using Ir(III) complex

**DOI:** 10.1038/s41598-025-19891-x

**Published:** 2025-10-15

**Authors:** Misaki Ochiai-Noguchi, Aoi Horikoshi, Seina Hirakata, Toshitada Yoshihara

**Affiliations:** 1 MIRAI Technology Institute, Shiseido Co., Ltd, 1-2-11, Takashima, Nishi-ku, Yokohama-shi, Kanagawa 220-0011 Japan; 2 Graduate School of Science and Technology, Gunma University, 1-5-1, Tenjin-cho, Kiryu-shi, Gunma 376-8515 Japan

**Keywords:** Oxygen, Iridium(III) complex, Phosphorescence lifetime, Differentiation, Keratinocyte, Skin, Biochemistry, Biological techniques, Cell biology, Physiology

## Abstract

Reconstructed human epidermis (RHE) is a useful experimental tool for evaluating the effects of various stimuli on the skin. Here, we present a method to visualize the partial pressure of oxygen in RHE with cellular-level spatial resolution by means of confocal phosphorescence-lifetime imaging microscopy (PLIM) with a cell-permeable phosphorescent probe, BTPDM1 (an iridium-based cationic lipophilic dye). Z-stack PLIM images of RHE revealed an oxygen partial pressure gradient in the direction of differentiation, with a decrease in oxygen levels from the basal layer to the cornified layer. FCCP (carbonyl cyanide p-(trifluoromethoxy)phenylhydrazone), an uncoupler of mitochondrial oxidative phosphorylation, significantly decreased *p*O₂, suggesting that changes in mitochondrial respiration may contribute to the O₂ concentration gradient in RHE. Antimycin A (Ant A), an inhibitor of mitochondrial respiration, decreased expression of the differentiation markers filaggrin and loricrin, indicating that mitochondrial respiration is essential for normal epidermal maturation. In 2D keratinocyte cultures, addition of calcium as a differentiation inducer led to an increase in mitochondrial oxygen consumption and oxidative phosphorylation. These results indicate that oxygen imaging is an effective method for evaluating not only mitochondrial respiration status, but also the differentiation state of reconstructed human epidermis.

**Supplementary Information:**

The online version contains supplementary material available at 10.1038/s41598-025-19891-x.

## Introduction

The skin is the largest organ of the human body and forms a functional and physical barrier between the skin and the external environment. The skin barrier plays an important role in preventing allergens and microorganisms from entering the human body, and a defective skin barrier is a key pathological feature in atopic dermatitis^1–3^. The epidermis is composed of several layers: the basal layer, the spinous layer, the granular layer and the cornified layer. The cornified layer is the outermost layer of the epidermis and represents the final stage of keratinocyte maturation and development. Keratinocytes in the basal layer of the epidermis are proliferative, but gradually lose their proliferative capacity and undergo programmed destruction as they mature in the epidermis. The keratinocytes that eventually differentiate and lose their nuclei are designated as keratinizing, and retain only keratin filaments embedded in a filaggrin matrix. The lipid envelope of these cells replaces the former keratinocyte plasma membrane, and the cells flatten and stack into layers interconnected by keratin desmosomes^4^.

In addition to these complex structural changes, the epidermis is also constantly renewed by active turnover. The epidermal progenitor cells are highly proliferative, metabolically active, and dependent on adenosine triphosphate (ATP) to meet their energy requirements. ATP is produced primarily in mitochondria by the process of oxidative phosphorylation (OXPHOS), which requires oxygen (O₂)^5^. Mitochondrial respiration utilizes a series of protein complexes in the inner membrane, collectively referred to as the electron transfer chain (ETC). These complexes sequentially transport electrons and protons across the intermembrane space, creating a proton gradient that supports ATP generation^6^. Impaired mitochondrial respiration and function have been reported to be associated with impaired normal differentiation of keratinocytes and aging^7–12^. Recently, multicolor two-photon imaging techniques have shown that energy metabolism in the epidermis changes from glycolysis to OXPHOS depending on differentiation status, suggesting that mitochondrial respiration is deeply involved in formation of the epidermis^13^.

**Fig. 1:**
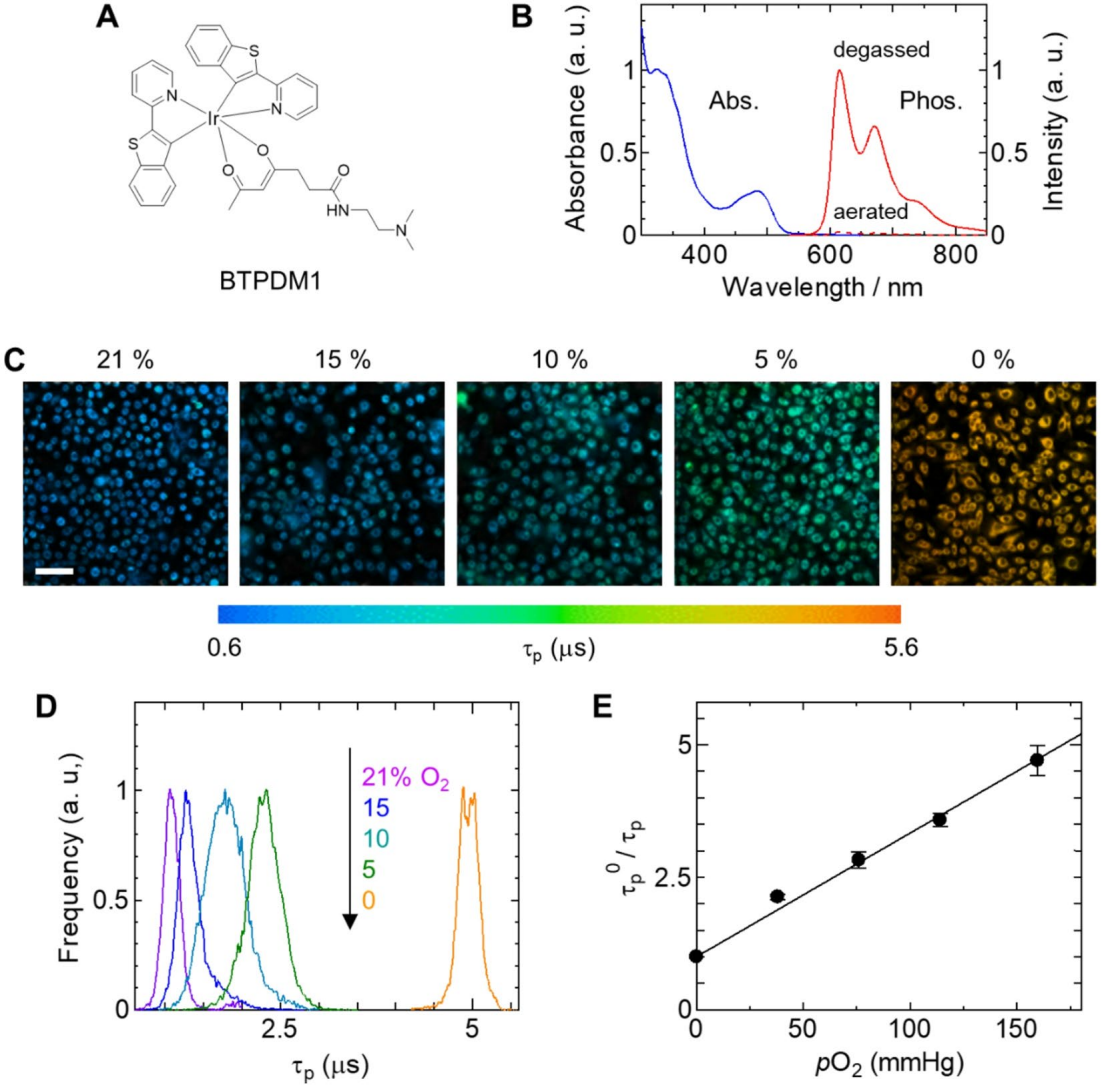
Characterization of intracellular O_2_ probes and quantification of O_2_ levels. (A) Chemical structure of BTPDM1. (B) Absorption and phosphorescence spectra of BTPDM1 in tetrahydrofuran. (C) PLIM images of keratinocytes stained with BTPDM1 under different conditions of *p*O₂ in an incubator. Cells were treated with Ant A (5–21% O_2_) and Na_2_SO_3_ (0% O_2_). Scale bar: 100 mm. (D) Distribution histograms of phosphorescence lifetime for the PLIM images in (C). (E) Stern–Volmer plots of τ_p_^0^/τ_p_ as a function of *p*O₂ for BTPDM1 partitioned into keratinocytes under different conditions of *p*O_2_ in an incubator. Error bars: S.D.

Oxygen is an essential element for the efficient production of ATP in mitochondria. It diffuses into tissues and cells through capillaries via diffusion gradients and serves as a substrate for many intracellular biochemical reactions, as well as mitochondrial ATP production^14,15^. To visualize tissue O₂ levels with high sensitivity and high stability, various phosphorescent metal complexes have been developed over the past few decades^16–19^. The compounds most commonly used as luminescent O₂ probes include Pt(II) and Pd(II) porphyrins, Ru(II) complexes, Pt(II) complexes, and Ir(III) complexes, which produce intense phosphorescence in the visible to near-infrared wavelength regions with reasonably long lifetimes (> 1.0 µs). O₂ probes targeting various biological tissues of interest have been developed by modifying these O₂-sensing luminophores. We recently developed a small-molecule O₂ probe based on the Ir(III) complex, BTPDM1 (Fig. 1A), which enables measurement of O₂ levels in hypoxic tumors^20,21^ as well as high-resolution O₂ imaging of the renal cortex^22^ and hepatic tissues^23^ in vivo, providing detailed insight into the distribution of tissue oxygen. In the present study, we established a technique for accurately visualizing oxygen distribution at the cellular level in epidermal tissue, which lacks blood vessels, and employed it to clarify the effects of changes in oxygen distribution on the formation of normal epidermis.

## Results

### Evaluation of oxygen probe BTPDM1 in keratinocytes

To measure intracellular partial pressure of oxygen (*p*O₂), we used BTPDM1 (Fig. 1A), an Ir(III) complex developed by Yoshihara et al.^21^ that emits red phosphorescence in degassed solution (Fig. 1B).

To quantify oxygen levels in tissues based on phosphorescence-lifetime measurements, a calibration curve is required. BTPDM1 has been reported to accumulate in lysosomes^21^, and the *p*O₂ dependence of phosphorescence lifetime in cells is expected to be different from that in solution. Therefore, the calibration curve was obtained by acquiring phosphorescence-lifetime imaging microscopy (PLIM) images of keratinocytes incubated with BTPDM1 under 21, 15, 10, 5, and 0% O_2_ conditions at 37 °C (Fig. 1C, D). We also investigated the probe toxicity in keratinocytes. Cell viability did not change with probe concentrations up to a maximum of 800 nM (Fig. S1). This result confirmed that the cytotoxicity of BTPDM1 is negligibly small under the conditions of the cell experiment, thus excluding any possible contributions of the probe’s cell toxicity to phosphorescence lifetime. Prior to PLIM measurements, 10 µM antimycin A (AntA) was added to the medium to suppress oxygen consumption by cellular respiration, and for experiments conducted under N_2_ saturated conditions, Na₂SO₃ (500 mM) was added to the medium to remove residual oxygen in the medium. The average lifetime across images was plotted against *p*O₂ according to the Stern–Volmer equation, τ_p_^0^/τ_p_ = 1+*k*_q_τ_p_^0^*p*O_2_. A linear relationship was obtained (Fig. 1E), and the *k*_q_ value of BTPDM1 was estimated to be 4.74 × 10^3^ mmHg^−1^ s^−1^ and τ_p_^0^ to be 4.94 µs. These values ​​were used to quantify the intracellular oxygen levels from the phosphorescence lifetime (τ_p_) according to the following equation, as derived from the Stern–Volmer equation.

$$pO_{2} = {\text{ }}1/k_{q} \left( {1/\tau _{p} - {\text{ }}1/\tau _{p} ^{0} } \right)$$

### Changes of *p*O2 in reconstructed human epidermis (RHE) along the z-axis

In this study, we used a commercially available RHE, EpiDerm (Mattek^24,25^), which was differentiated from human foreskin-derived epidermal keratinocytes. It has similar properties to human skin, and is used as a substitute for human skin in various evaluations, such as skin irritation tests^26–28^. In this study, we used a model with a culture period 3 days shorter than that of regular EpiDerm, which has an immature cornified layer. On Day 3, the third day after delivery, the cornified layer is thin, while by Day 8, the cornified layer has differentiated and matured to form multiple layers (Fig. S2A). In addition, filaggrin, loricrin, and keratin 1, which are indicators of final differentiation of keratinocytes and are expressed in the granular layer, are more strongly expressed on Day 8 than on Day 3, indicating that the formation and differentiation state of the cornified layer depends on the duration of culture (Fig. S2B, C).

**Fig. 2:**
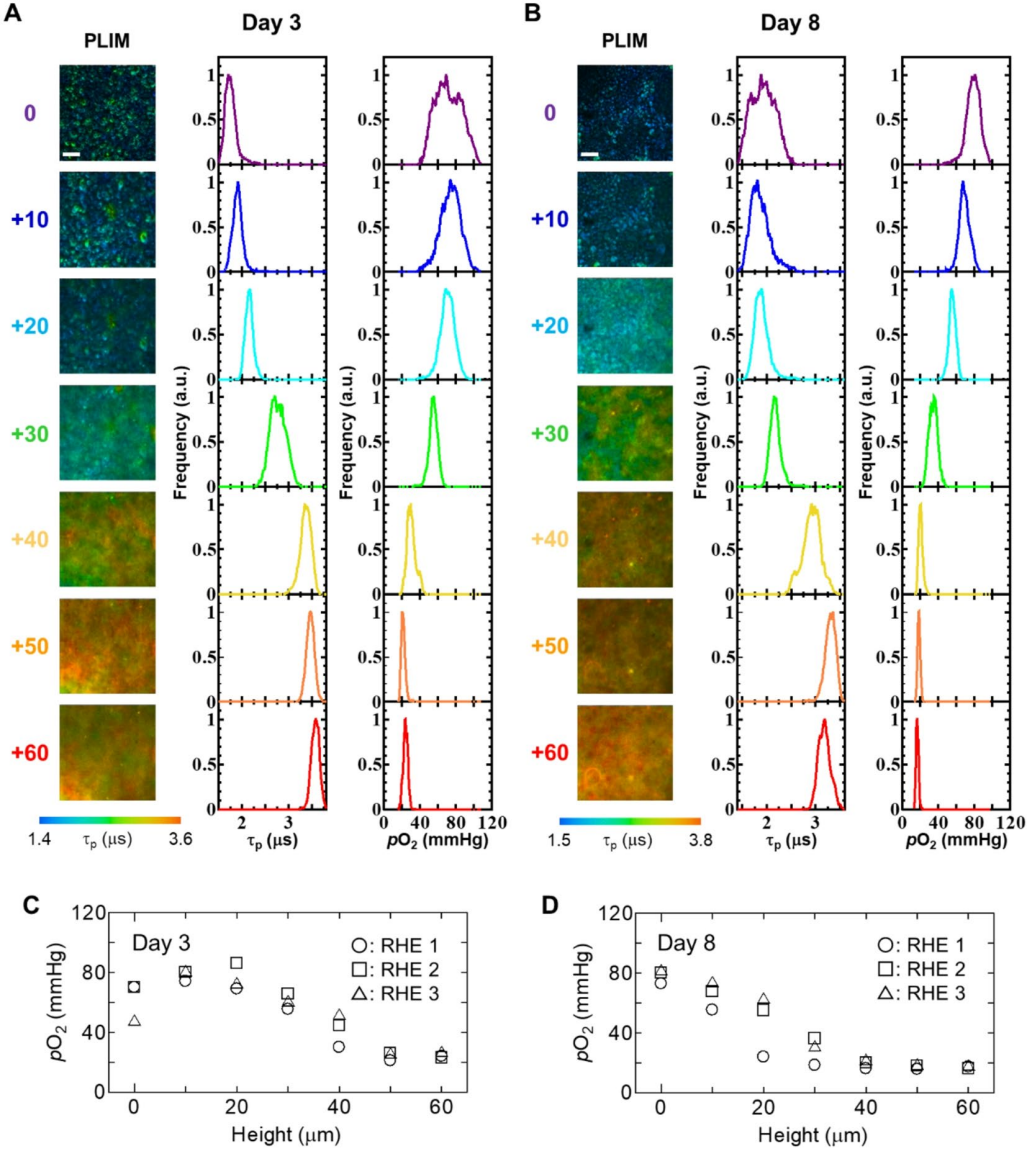
Changes in *p*O₂ in RHE along the z-axis. (A, B) Z-stacked PLIM images of RHE, and their distribution histograms of phosphorescence lifetime τ_p_ (left) and *p*O₂ (right). Scale bar: 100 mm. (C, D) Changes in *p*O₂ by field of view. Day 3 (A, C). Day 8 (B, D).

BTPDM1 has excellent cell permeability, and therefore may suitable for visualization of oxygen distribution in RHE to elucidate the physiological oxygen status in the skin. So, we first acquired z-direction PLIM images of RHE after incubation for 48 h with 5 µM BTPDM1. At this time point, the probe did not induce apoptosis, as confirmed by TUNEL assay (Fig. S3A, B). The point where the structure could be observed was set as 0, and the phosphorescence intensity and PLIM images were acquired in the vertical z direction (Fig. S4, Fig. 2A, B). It has been reported that the size of keratinocytes increases during differentiation^29^. We also observed that the size of the cells increased toward the cornified layer. *p*O₂ distribution was derived from τ_p_^0^ and *k*_q_ values obtained in keratinocytes and PLIM images. Z-stack PLIM images of RHE showed a decrease in oxygen levels from the basal layer to the cornified layer. Furthermore, comparison of the images acquired on Day 3 and Day 8 showed a significant decrease in *p*O₂ toward the cornified layer on Day 8 (Fig. 2C, D).

### *p*O2 changes within RHE in response to mitochondrial respiration

**Fig. 3:**
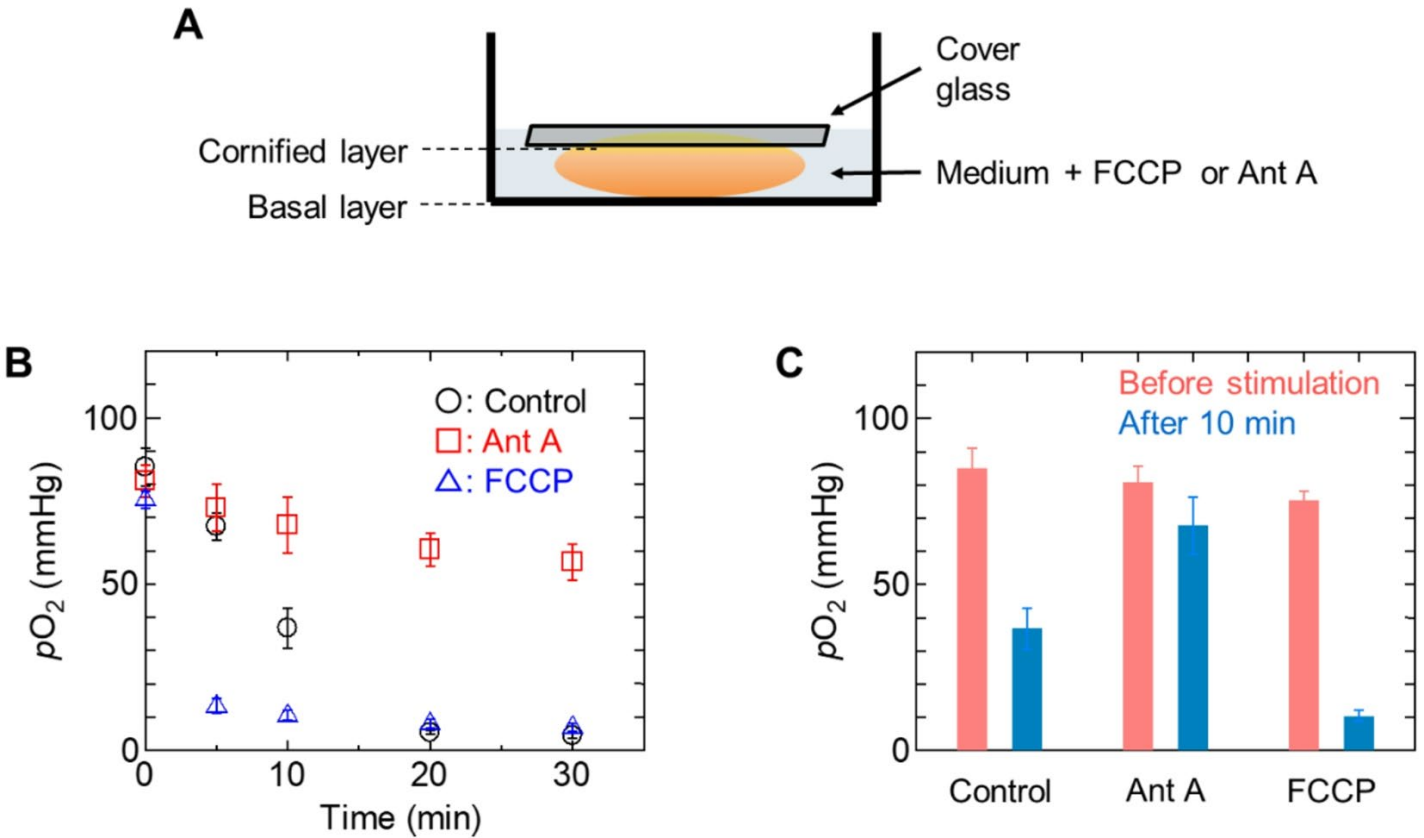
Imaging of the oxygen status of RHE in the presence of FCCP or Ant-A. (A) Schematic representation of RHE in media containing FCCP or Ant-A. (B) Variations of *p*O_2_ for the PLIM images shown in Figures S3, S4 and S5. (C) The *p*O_2_ values before stimulation and at 10 min after addition of FCCP or Ant-A.

Next, we investigated whether BTPDM1 could visualize changes in the oxygen state within RHE by modulating metabolic processes using FCCP (carbonyl cyanide p-(trifluoromethoxy)phenylhydrazone) and Ant A. FCCP is an inhibitor of oxidative phosphorylation in mitochondria and inhibits ATP synthesis by transporting protons across the membrane, thereby increasing the rate of oxygen consumption. Ant A inhibits the mitochondrial electron transport chain from cytochrome b to cytochrome c1 and inhibits oxygen consumption. Experiments were performed by placing a cover glass over RHE to prevent the epidermis from floating out of the medium (Fig. 3A). Since the cover glass inhibits the uptake of oxygen from air, *p*O₂ gradually decreased in the control as the oxygen consumption by mitochondria reduced the level of oxygen in the cells (Fig. 3B). When the *p*O₂ levels before and 10 min after the addition of each modulator were compared, no significant change in *p*O₂ was observed when Ant A was added, while a significant decrease in *p*O_2_ was observed when FCCP was added (Fig. 3C). Metabolic stimulation of RHE with FCCP and Ant A also significantly altered the PLIM images (Fig. S5, S6, S7). Observations up to 30 min after the addition of Ant A showed little change in τ_p_ or *p*O₂, whereas a rapid increase in τ_p_ and a decrease in *p*O₂ were observed within 10 min after the addition of FCCP. These results suggest that the change in *p*O₂ from basal to stratum corneum in RHE may be caused by changes in mitochondrial respiration.

### Mitochondrial respiration is essential for normal epidermal differentiation

**Fig. 4:**
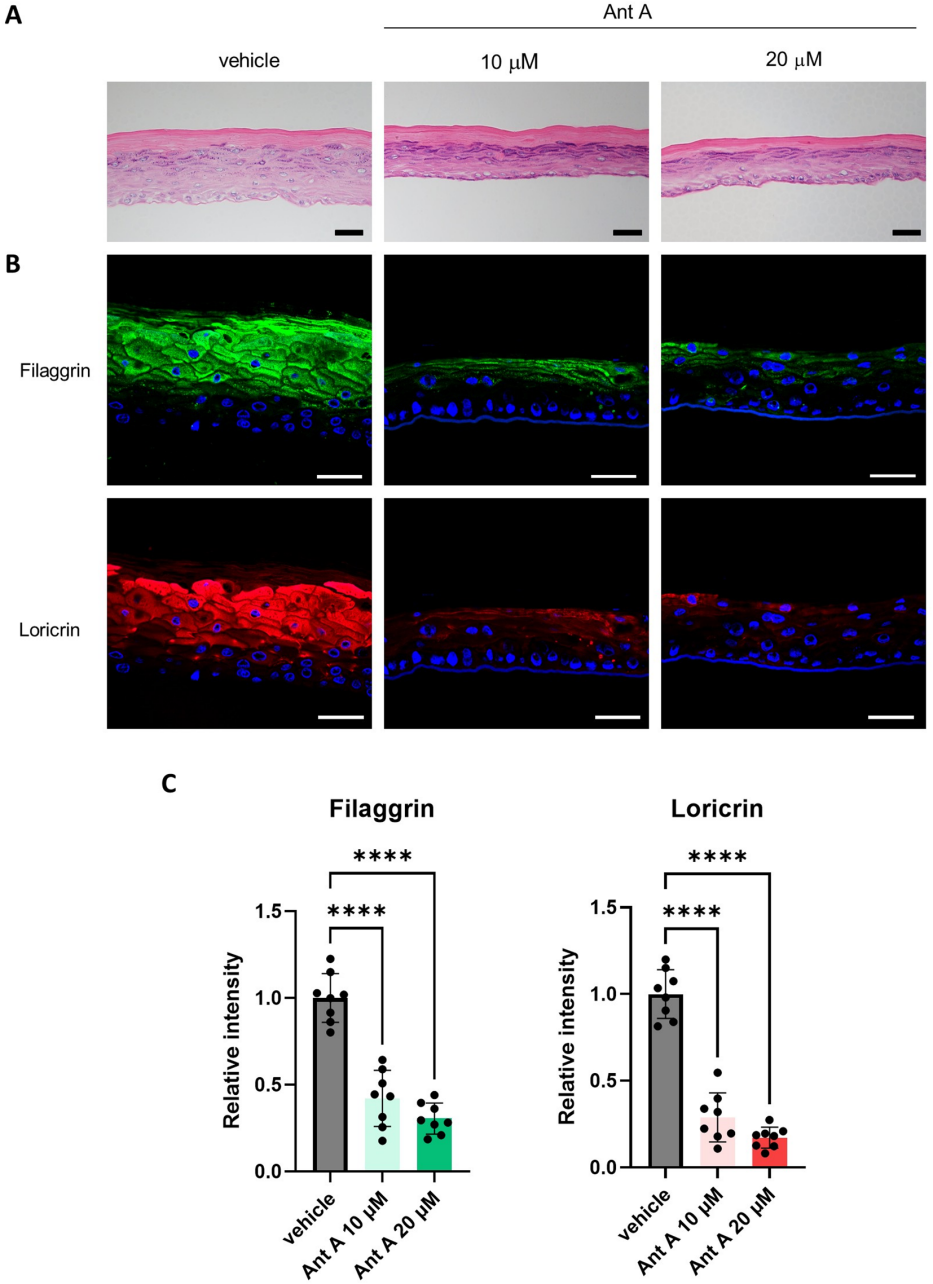
Effects of Ant-A on epidermal construction and differentiation. (A) HE staining of reconstructed human epidermis. Scale bar indicates 50 μm (B) Immunofluorescence staining of RHE showing expression of filaggrin and loricrin. Scale bar indicates 50 μm. (C) Relative intensity of filaggrin and loricrin staining. Bars and lines represent mean ± SD. ****: *p* < 0.0001.

To confirm the effect of mitochondrial respiration on epidermal differentiation, RHE was cultured in medium containing Ant A. On day 5, examination of the formation and differentiation of the cornified layer by means of HE staining showed that the epidermis was generally thinner and the living layer was reduced (Fig. 4A). Next, the expression of terminal differentiation markers, filaggrin and loricrin, in the epidermis was examined by immunofluorescence staining. Expression of these markers was significantly decreased by Ant A compared to the vehicle control (Fig. 4B). No concentration dependence was observed (Fig. 4C). Thus, inhibition of mitochondrial respiration inhibited normal epidermal differentiation.

### Mitochondrial oxygen consumption rate (OCR) changes depending on the differentiation state of keratinocytes

**Fig. 5:**
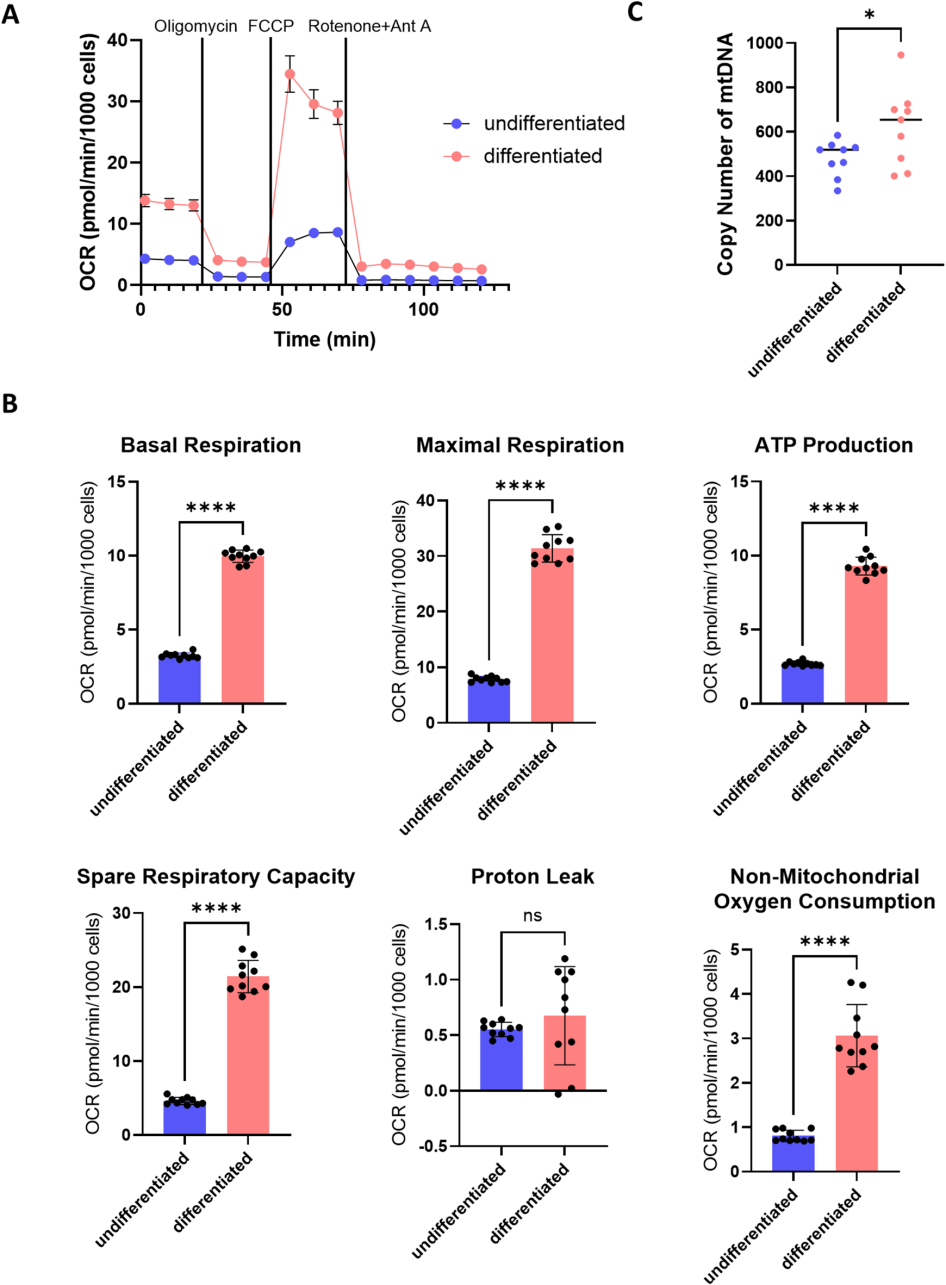
Comparison of OCR and copy number of mtDNA in undifferentiated and differentiated keratinocytes. (A) Seahorse assay for OCR in undifferentiated keratinocytes and differentiated keratinocytes. (B) Quantification of basal respiration, maximal respiration, ATP production, spare respiratory capacity, proton leakage and non-mitochondrial oxygen consumption. (C) Copy number of mtDNA in undifferentiated keratinocytes and differentiated keratinocytes. Bars and lines represent mean ± SD. *: *p* < 0.05, ****: *p* < 0.0001.

Next, we measured the OCR of keratinocytes in 2D culture to examine whether mitochondrial respiration changes depending on the differentiation state in this condition, as well as in RHE. Differentiation of keratinocytes is induced by adding high concentrations of calcium to the culture medium^30,31^. Therefore, we cultured keratinocytes for 48 h in the presence of 1.5 mM calcium and measured mitochondrial OCR. Keratinocyte differentiation was assessed in terms of the mRNA expression of filaggrin, loricrin, and keratin 1 (Fig. S8). OCR of keratinocytes was measured by means of the Seahorse assay, which can assess various mitochondrial bioenergetic parameters by incorporating different mitochondrial inhibitors with specific and distinct functions^32^. Oligomycin inhibits ATP synthase (complex V) and is injected first in the assay following basic measurements. Oligomycin decreases the flow of electrons through the electron transport chain (ETC), resulting in a decrease in mitochondrial respiration or OCR. This decrease in OCR affects cellular ATP production. The uncoupler FCCP is injected after oligomycin. As a result of the collapse of the proton concentration gradient, electron flow through the ETC is unimpeded and oxygen consumption by complex IV reaches a maximum. The OCR stimulated by FCCP can be used to estimate the spare respiratory capacity, defined as the difference between maximal and basal respiration. Pre-respiratory capacity is a measure of the cell’s ability to respond to increased energy demand or stress. The third injection is a mixture of rotenone, a complex I inhibitor, and Ant A, a complex III inhibitor. This combination stops mitochondrial respiration and allows estimation of non-mitochondrial respiration. Basal respiration, maximal respiration, ATP production, and spare respiratory capacity were upregulated in differentiated cells compared with undifferentiated cells (Fig. 5A, B). Keratinocytes underwent mitochondrial OXPHOS changes upon differentiation, suggesting increased oxygen consumption. It has been reported that mitochondrial OXPHOS and biosynthesis change upon differentiation from stem cells^33–35^. Therefore, to determine whether mitochondrial biosynthesis also changes in keratinocytes upon differentiation, we measured mtDNA copy number. The mtDNA copy number was increased in differentiated cells compared to undifferentiated cells (Fig. 5C). This suggests that mitochondrial biosynthesis may change with keratinocyte differentiation, which may be one of the reasons why metabolism changes with differentiation.

## Discussion

We first verified that BTPDM1 exhibits appropriate photophysical properties as an O_2_ probe for RHE. It has been reported that the metabolic gradient along the z-axis of the epidermis, determined by measuring NADH and FAD using multicolor two-photon imaging technology, shifts from an energy metabolism dominated by glycolysis in basal cells to an OXPHOS-dominated metabolism in differentiated cells^13^. The oxygen gradient in RHE observed in our study is consistent with these findings. As differentiation from the basal to the granular layer progresses, the shift from glycolysis-dominated energy metabolism to OXPHOS results in a decrease in intracellular *p*O₂ and the formation of an oxygen gradient in the epidermis.

In the skin, capillaries exist in the papillary layer of the dermis just below the epidermis, supplying oxygen to the epidermis and dermis^36^. Oxygen diffuses from the atmosphere to a depth of 266–375 μm in the skin tissue, and the oxygen concentration increases from the basal layer to the cornified layer and from the basal layer to the dermis due to the combined effects of diffusion of oxygen from the atmosphere through the epidermis and oxygen supply from capillaries^37^. The oxygen concentration gradient is reported to be high from the basal layer to the cornified layer and from the basal layer to the dermis^38^. On the other hand, a study of the *p*O₂ distribution in the epidermis of the nail contour using oxygen-sensitive microelectrodes found that *p*O₂ increases with depth from the skin surface to the dermis^39^. Our previous studies have shown that *p*O₂ decreases from the outside to the inside of spheroids of HT-29 cells^23^. Furthermore, we have shown that phosphorescence emission is maintained in the center of the cells when *p*O₂ is measured by placing a cover glass over the monolayer cells^21^. Considering these results, it seems plausible that the cornified layer, which consists of several layers of cells, suppresses most of the diffusion of oxygen from the external environment. The study of oxygen diffusion from the atmosphere into the skin was conducted after the cornified layer had been removed by 10 tape strippings prior to the measurements. In other studies, it was found that the granular layer becomes exposed in some parts of the epidermis after 15 tape strippings^40^. Thus, stripping may have facilitated the diffusion of oxygen into the epidermis by removing the cornified layer. On the other hand, the RHE used in this study lacks capillaries in its tissue structure and is known to have an incomplete cornified layer compared to human skin^41^. Considering the possibility that the primary oxygen supply to the skin is provided by capillaries and that the cornified layer suppresses the influx of oxygen from the external environment, it is challenging to fully replicate the oxygen conditions within human skin using the RHE model. This remains an issue for future research.

Although the nature of the concentration gradient of oxygen in the epidermis remains controversial, *p*O₂ imaging has the potential not only to visualize oxygen consumption due to mitochondrial respiration, but also to reveal the differentiation state of the skin. Psoriasis is a well-known skin disease in which epidermal differentiation is impaired. It is a chronic inflammatory autoimmune skin disease influenced by genetic and various environmental factors, and lesions are characterized by excessive proliferation of keratinocytes, leading to epidermal hyperplasia^42,43^. Single-cell RNA sequence data analysis revealed that the expression of glucose transporter SLC2A1 and lactate transporter SLC16A1, which are involved in lactate uptake, is significantly increased in undifferentiated keratinocytes in psoriasis, leading to increased glucose uptake for glycolysis and increased lactate uptake for TCA cycle activation^44^. Accurately capturing metabolic changes and differentiation state in the epidermis in three dimensions by measuring *p*O₂ should provide new insights into intracellular gene and protein changes as well as actual mitochondrial respiratory and metabolic changes in psoriasis.

Alterations in energy metabolism occur not only in diseases but also in aging. Age-dependent accumulation of ROS in keratinocytes leads to a metabolic shift from OXPHOS to anaerobic glycolysis, and aging is accompanied by mutations and deletions of mtDNA, leading to a steady decline in mitochondrial function^9,11^. Our findings suggest that mtDNA increases with differentiation and mitochondrial biogenesis increases. It is possible that mitochondrial function is impaired due to the accumulation of mtDNA damage with age, and differentiation fails to proceed normally because the impaired mitochondrial function is not able to respond to the increased oxygen demand that accompanies differentiation. The levels of epidermal permeability barrier proteins, including filaggrin, loricrin, and other late cornified proteins, are significantly reduced with aging^45–47^. Restoring energy production through OXPHOS could be one strategy to resolve differentiation failure associated with aging. In this context, imaging oxygen consumption and oxygen concentration gradients in aging skin should lead to a better understanding of the metabolic changes associated with aging.

## Conclusions

We used the previously synthesized iridium (III) complex BTPDM1 as a tool for imaging *p*O₂ in RHE. BTPDM1 has excellent photophysical properties as an O₂ probe in aqueous media, with a quenching rate constant of 4.74 × 10³ mmHg⁻¹ s⁻¹ and τp0 of 4.94 µs at 37℃ in keratinocytes. *p*O₂ in RHE was calculated using the parameters measured in keratinocytes. PLIM images of RHE incubated with BTPDM1, acquired in the z direction, showed that the O₂ level decreased from 80 mmHg near the basal layer to 30 mmHg towards the cornified layer. Furthermore, there was a significant decrease in *p*O₂ towards the cornified layer on Day 8 compared with Day 3. A significant decrease in *p*O₂ was observed upon addition of FCCP, suggesting that changes in mitochondrial respiration may cause the O₂ concentration gradient in RHE. The addition of Ant A to RHE resulted in decreased expression of filaggrin and loricrin, indicating that mitochondrial respiration is essential for normal epidermal maturation. We found that OCR increased in keratinocytes induced to differentiate by high concentrations of calcium in 2D culture. Furthermore, the increase in mitochondrial DNA copy number following induction of differentiation suggested that mitochondrial biogenesis and respiration might be enhanced as differentiation progresses. Overall, our results indicate that imaging *p*O₂ in RHE enables the evaluation of not only mitochondrial respiration status, but also differentiation status.

## Materials and methods

### Materials

Antimycin A from *Streptomyces* sp. (Ant A) and carbonyl cyanide 4-(trifluoromethoxy)phenylhydrazone (FCCP, 98%) were purchased from Sigma-Aldrich (MO, USA).

### Cells and culture

Normal human epithelial keratinocytes were purchased from Kurabo (Osaka, Japan) and cultured in HuMedia-KG2 (Kurabo, Osaka, Japan). Under undifferentiated conditions, keratinocytes were cultured to a confluency of 80 to 90% at the time of measurement. For differentiation induction, keratinocytes were seeded to over-confluency, and then cultured for 48 h from the following day in a medium containing 1.5 mM CaCl_2_. Culture flasks were incubated at 37 °C in a humidified atmosphere with 5% CO_2_. RNA was extracted from each sample for quantitative PCR analysis. For PLIM imaging, cells and reconstructed human epidermis were cultured on glass-based dishes, and the medium was changed to FluoroBrite DMEM (A1896701, Thermo Fisher Scientific, MA, USA) prior to the measurements. BTPDM1 was added to the medium at a concentration of 500 nM 2 h before observation. To assess the toxicity of BTPDM1, cells were incubated for 2 to 24 with various concentrations of BTPDM1 and cell viability was assessed using alamarBlue Cell Viability Reagent (DAL1025, Thermo Fisher Scientific, MA, USA).

### Reconstructed human epidermis

The RHE model (EPI-201) and medium (EPI-201-DM, EPI-100-NMM-3) were purchased from MatTek Corp. (Ashland, MA, USA). The culture medium was changed every other day. For the first 3 days, the culture medium was EPI-201-DM. After 3 days the culture medium was EPI-100-NMM-3. At 3 days, 5 days and 8 days after the start of culture, samples were fixed in cold acetone (AMeX procedure) and embedded in paraffin for hematoxylin and eosin (H&E) staining and immunohistochemical analysis. PLIM measurement was conducted at 3 days and 8 days after the start culture. BTPDM1 was added to the medium at a concentration of 5 µM 48 h before observation.

### PLIM imaging of reconstructed human epidermis

Confocal phosphorescence intensity and lifetime images were acquired with an inverted fluorescence microscope (IX73, Olympus) equipped with a confocal scanning system (DCS-120, Becker & Hickl) and a picosecond diode laser (BDL-SMC, Becker & Hickl; wavelength: 488 nm, pulse width: 40–90 ps, repetition rate: 50 MHz)^23^. Emission signals were detected using a hybrid detector module (HPM-100-40, Becker & Hickl). Fluorescence and phosphorescence decay curves were determined using a time-correlated, single-photon counting unit and a multichannel scaler unit (Simple-Tau-150-DX, Becker & Hickl), respectively. Scanning was performed at a frame time of 1.11 s, corresponding to a pixel dwell time of 64.0 µs. The total acquisition time was 56 s, corresponding to an accumulation of 50 frames. Phosphorescence-lifetime images were generated by analyzing the decay curves for each pixel using SPCImage data analysis software (Becker & Hickl).

### Quantitative real-time RT-PCR

The total RNA from cultured keratinocytes was isolated using a Qiagen Rneasy mini kit (Qiagen, Tokyo, Japan), and cDNA was synthesized using a SuperScript VILO cDNA Synthesis Kit (11754250, Thermo Fisher Scientific, MA, USA). Expression of filaggrin, loricrin and keratin 1 genes in the epidermis was analyzed by quantitative PCR using Platinum SYBR Green qPCR superMix-UDG (11744500, Invitrogen Japan, Tokyo, Japan). The PCR reactions were performed on StepOnePlus (Thermo Fisher Scientific, MA, USA). The primer sequences used were as follows: RPLP0, 5′-CCTTCTCCTTTGGGCTGGTCATCCA-3′ (forward) and 5′-CAGACACTGGCAACATTGCGGACAC-3′ (reverse); filaggrin, 5′-GGCAAATCCTGAAGAATCC-3′ (forward) and 5′-TGCTTTCTGTGCTTGTGTCC-3′ (reverse); loricrin, 5′-GGGCACCGATGGGCTTAG-3′ (forward) and 5′-GGTAGGTTAAGACATGAAGGATTTGC-3′ (reverse); keratin 1, 5′-GTTCCAGCGTGAGGTTTGTT-3′ (forward) and 5′-TAAGGCTGGGACAAATCGAC-3′ (reverse).

### Immunohistochemistry

Samples were fixed with cold acetone, embedded in paraffin, and sectioned at 4 μm for hematoxylin and eosin (H&E) staining and immunostaining. For immunostaining, antibodies to filaggrin (sc-66192, Santa Cruz Biotechnology, TX, USA), loricrin (905104, Biolegend, CA, USA), and keratin 1 (sc-376224, Santa Cruz Biotechnology, TX, USA) were used as primary antibodies. The secondary antibodies used were Alexa Fluor 594 donkey anti-rabbit IgG (H + L) (A21207, Invitrogen, CA, USA) for loricrin, and Alexa Fluor 488 donkey anti-mouse IgG (H + L) (A21202, Invitrogen, CA, USA) for filaggrin and keratin 1. The nucleus was stained using VECTASHIELD Mounting Medium with DAPI (H-1200, Vector laboratories, CA, USA).Samples were observed with a STELLARIS 5 confocal scanner unit (LEICA Microsystems, Wetzlar, Germany). The excitation and emission for each antibody were 496/501–739 nm for Alexa Fluor 488 and 594/601–750 nm for Alexa Fluor 594. 380/400–619 nm for DAPI. To evaluate fluorescence intensity, 4–6 images were randomly selected from each condition, and the areas were measured with ImageJ 1.54p software.

### Seahorse assay

Oxygen consumption rate (OCR) was measured using an Agilent Seahorse XFe24 Analyzer and Cell Mito Stress Test Kit (103015-100, Agilent Technology, CA, USA). Keratinocytes were seeded at 2 × 10^4^ (undifferentiated) or 6 × 10^4^ (differentiated) cells per plate. The following day, 1.5 mM CaCl_2_ was added to the culture medium of differentiated cells. Before the assay, the cartridge sensor was hydrated overnight in Seahorse Bioscience XFe24 Calibration buffer (Agilent Technology, CA, USA) at 37 °C without CO_2_. On the day of the assay, XF DMEM Medium (103680-100, Agilent Technology, CA, USA) was added. The cells were incubated at 37 °C without CO_2_ for 1 h and transferred to the XFe24 analyzer. OCR was monitored under basal conditions and measured after injection of oligomycin (1 µM), FCCP (1 µM) or rotenone/antimycin A (0.5 µM). The results were analyzed using Seahorse XFe24 software.

### Apoptosis assay

TUNEL staining was performed with a TACS 2 TdT-DAB In Situ Apoptosis Detection Kit (4810-30-K, R&D Systems, MO, USA) according to the manufacturer’s instructions.

### MtDNA copy number

Total DNA was extracted from cell samples using the NucleoSpin Tissue kit (740952.50, Macherey-Nagel, Dueren, Germany) with proteinase K and RNase treatment, according to the manufacturer’s instructions. To quantify mtDNA copy number, real-time PCR was performed using a Mitochondrial DNA Copy Number Kit (MCN1, Detroit R&D, MI, USA), according to the manufacturer’s instructions.

### Quantification and statistical analysis

#### Statistics

The statistical significance of differences among three or more groups was determined by ANOVA with Scheffé’s method. Student’s *t* test was used to determine the significance of differences between two groups. A *p*-value < 0.05 was considered significant.

## Supplementary Information

Below is the link to the electronic supplementary material.

Supplementary Material 1

## Data Availability

The datasets generated during and/or analyzed during the current study are available from the corresponding author on reasonable request.
