# Supplementary material for: Visualization of oxygen profile in reconstructed human epidermis by phosphorescence-lifetime imaging microscopy using Ir(III) complex
Source: Sci Rep. 2025 Oct 15;15:36008. doi: 10.1038/s41598-025-19891-x (PMC12528490; doi:10.1038/s41598-025-19891-x)
Supplement: Supplementary file 1 — Supplementary Material 1 [file 41598_2025_19891_MOESM1_ESM.docx]

**Supplementary Figure 1**. **Evaluation of the effect of BTPDM1 on cell viability**

Cell viability evaluation using the AlamarBlue assay. Cells were incubated with various concentrations of BTPDM1 for 2 to 24 h, and fluorescence was measured at Ex 560 nm and Em 590 nm.


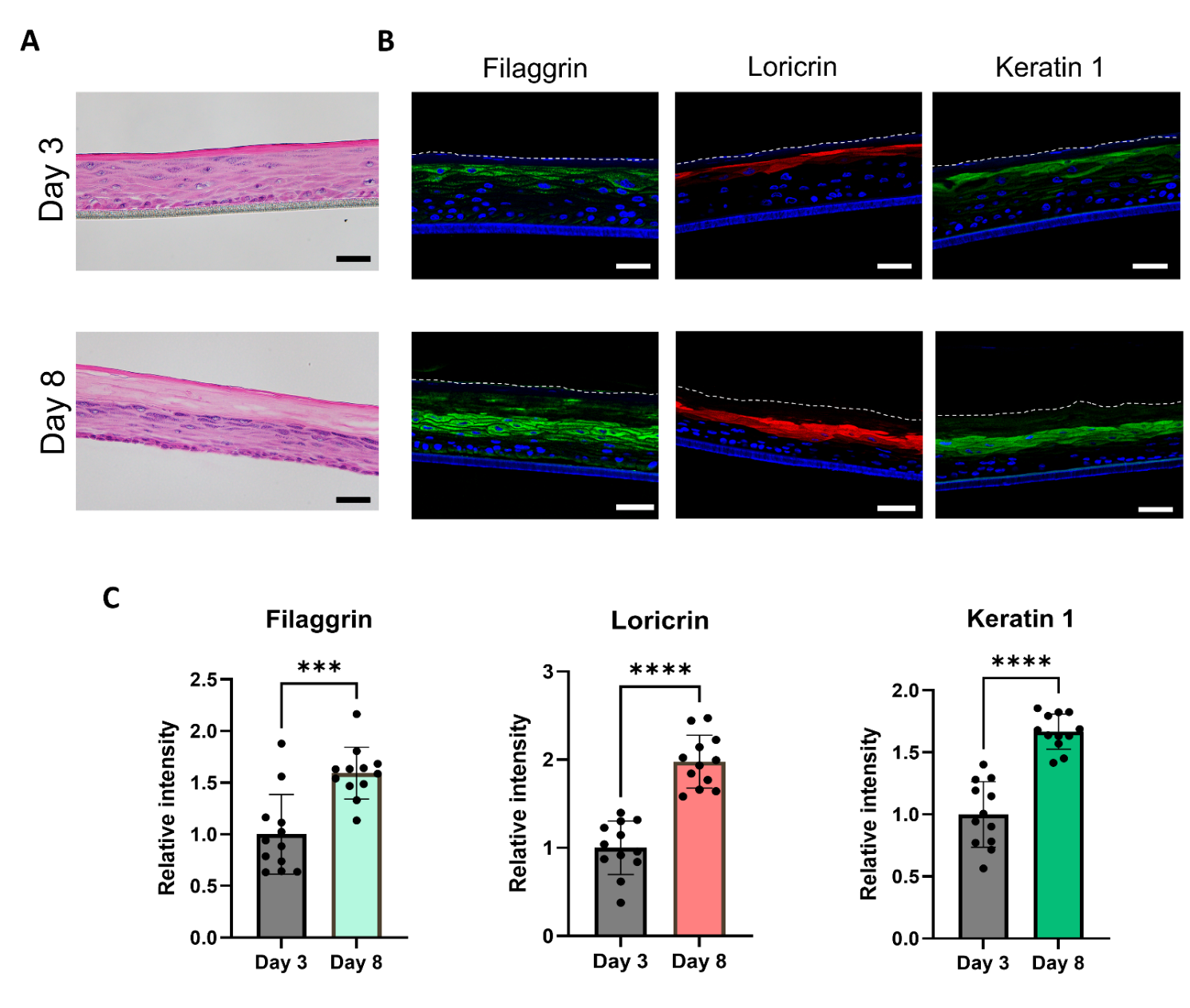


**Supplementary Figure 2. Changes of maturation state in reconstructed human epidermis (RHE).**

(A) HE staining of RHE. Scale bar indicates 50 µm (B) Immunofluorescence staining of RHE. Expression of filaggrin, loricrin and keratin 1. Scale bar indicates 50 µm. (C) Relative intensity of filaggrin, loricrin and keratin 1 staining. Bars and lines represent mean ± SD. ***: p < 0.0002, ****: p < 0.0001.


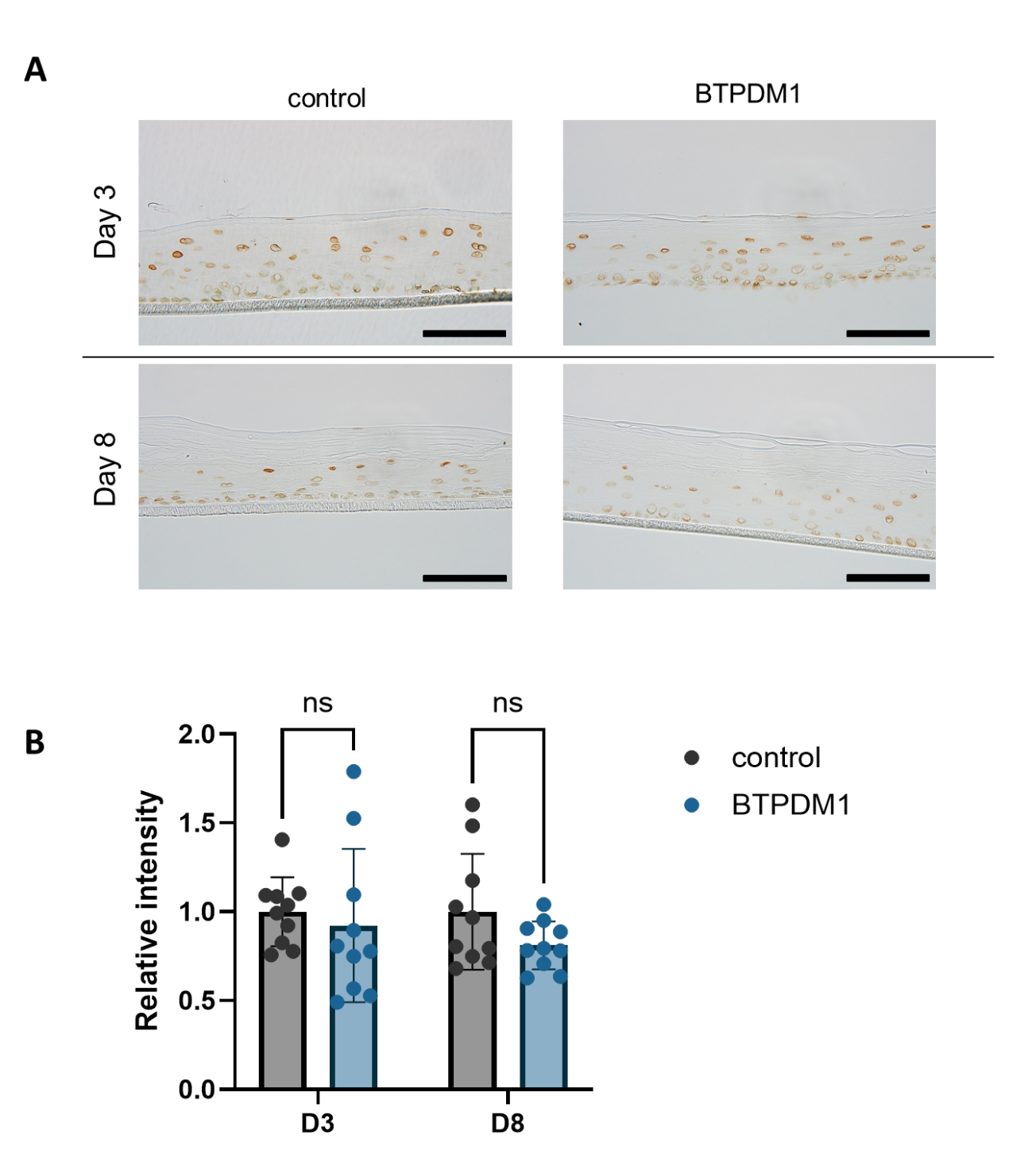


**Supplementary Figure 3. Evaluation of the effect of BTPDM1 on cell damage**

(A) Apoptosis assay by TUNEL staining of reconstructed human epidermis with or without 10 µM BTPDM1. Scale bar indicates 100 µm. (B) Relative intensity of TUNEL staining. Bars and lines represent mean ± SD.


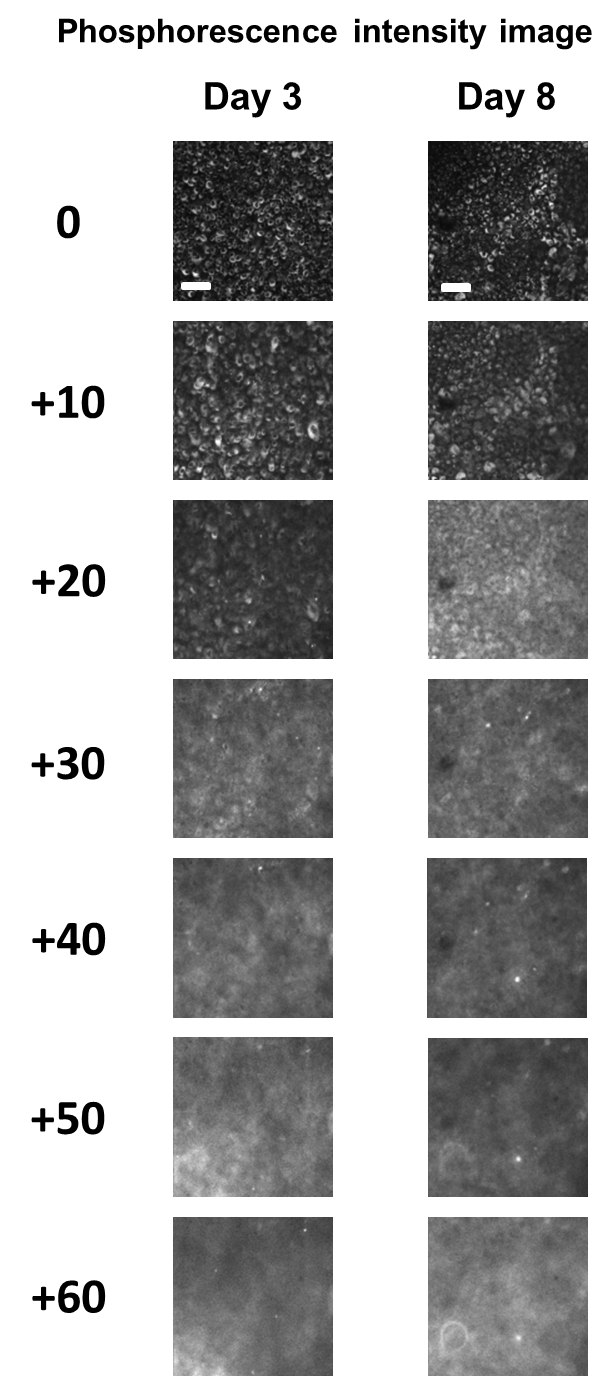


**Supplementary Figure 4. Z-stacked phosphorescence intensity images of RHE. Scale bar: 100 mm.**


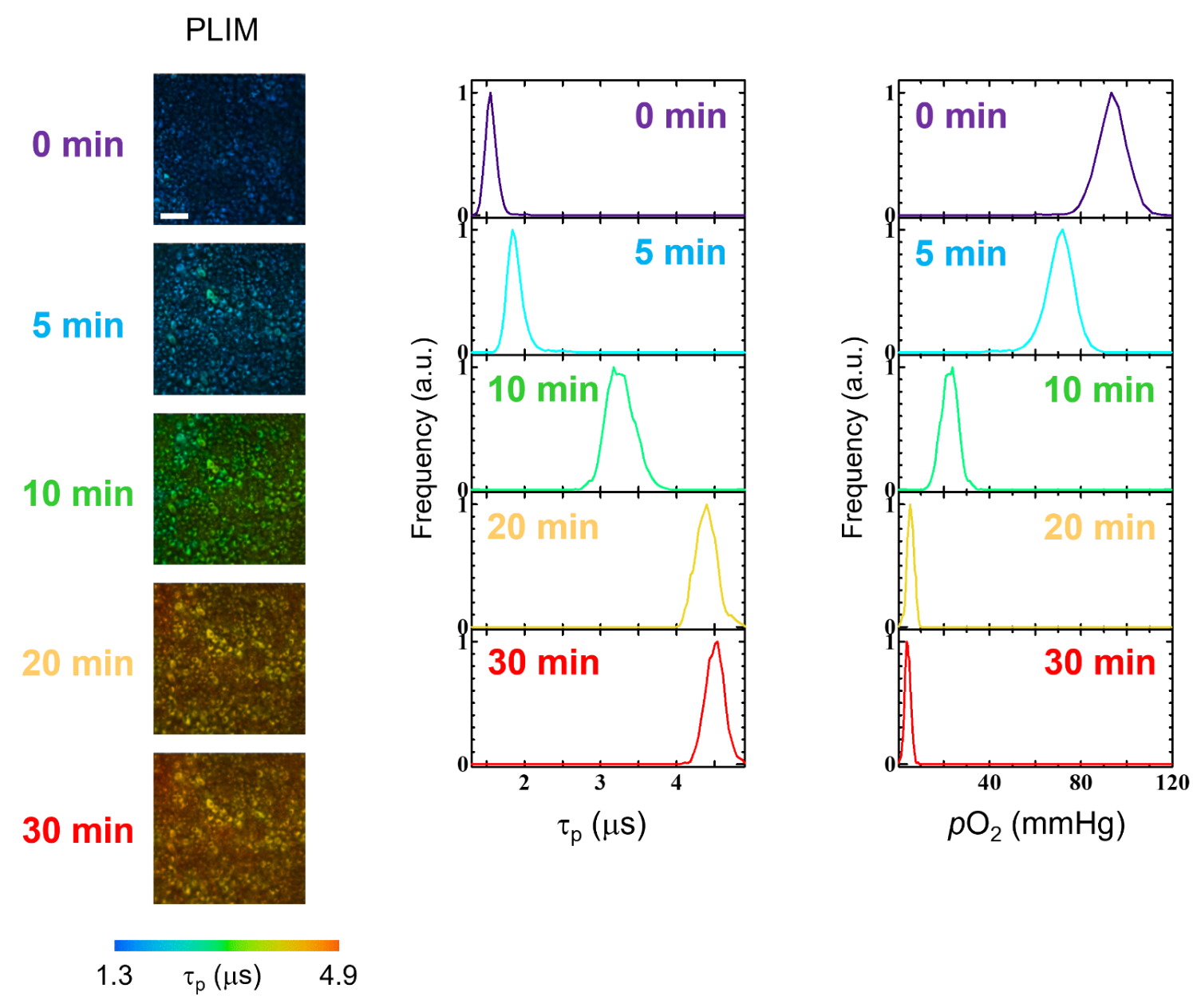


**Supplementary Figure 5. PLIM images of control RHE stained with BTPDM1, and their distribution histograms of phosphorescence lifetime and pO2.**

**
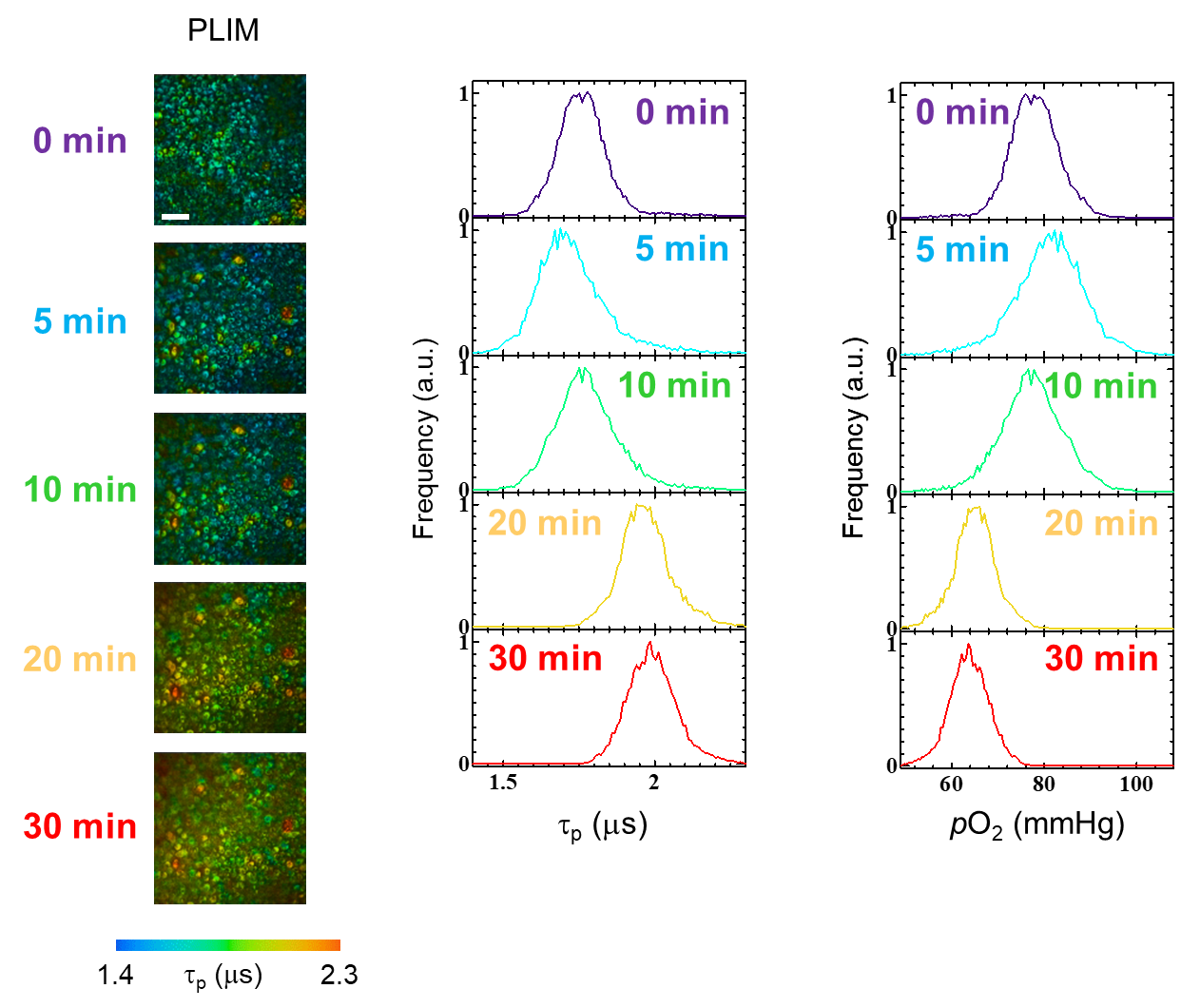
**

**Supplementary Figure 6. PLIM images of 20 µM Ant A treated RHE stained with BTPDM1, and their distribution histograms of phosphorescence lifetime and pO2**


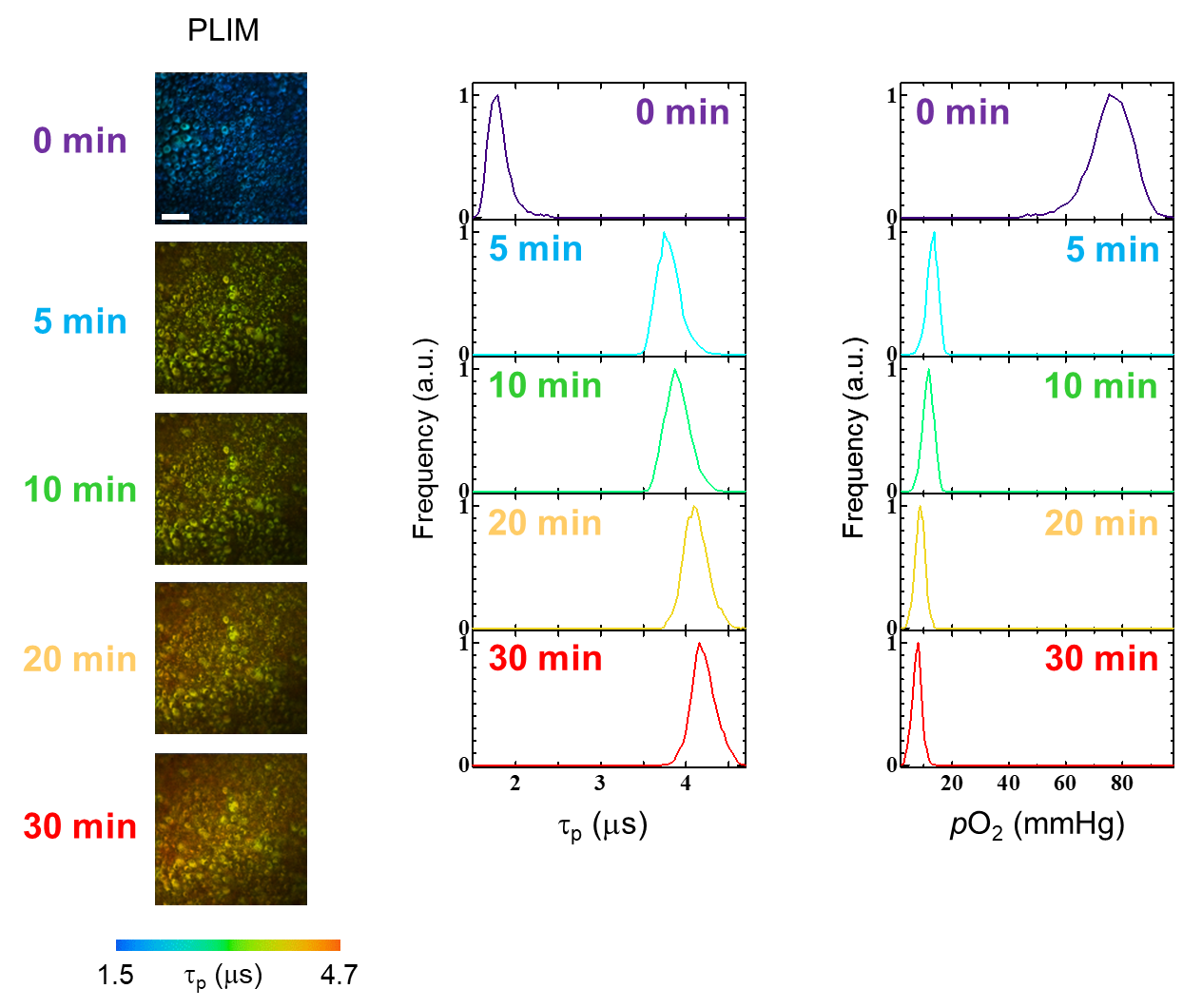


**Supplementary Figure 7. PLIM images of 10 µM FCCP treated RHE stained with BTPDM1, and their distribution histograms of phosphorescence lifetime and pO2.**


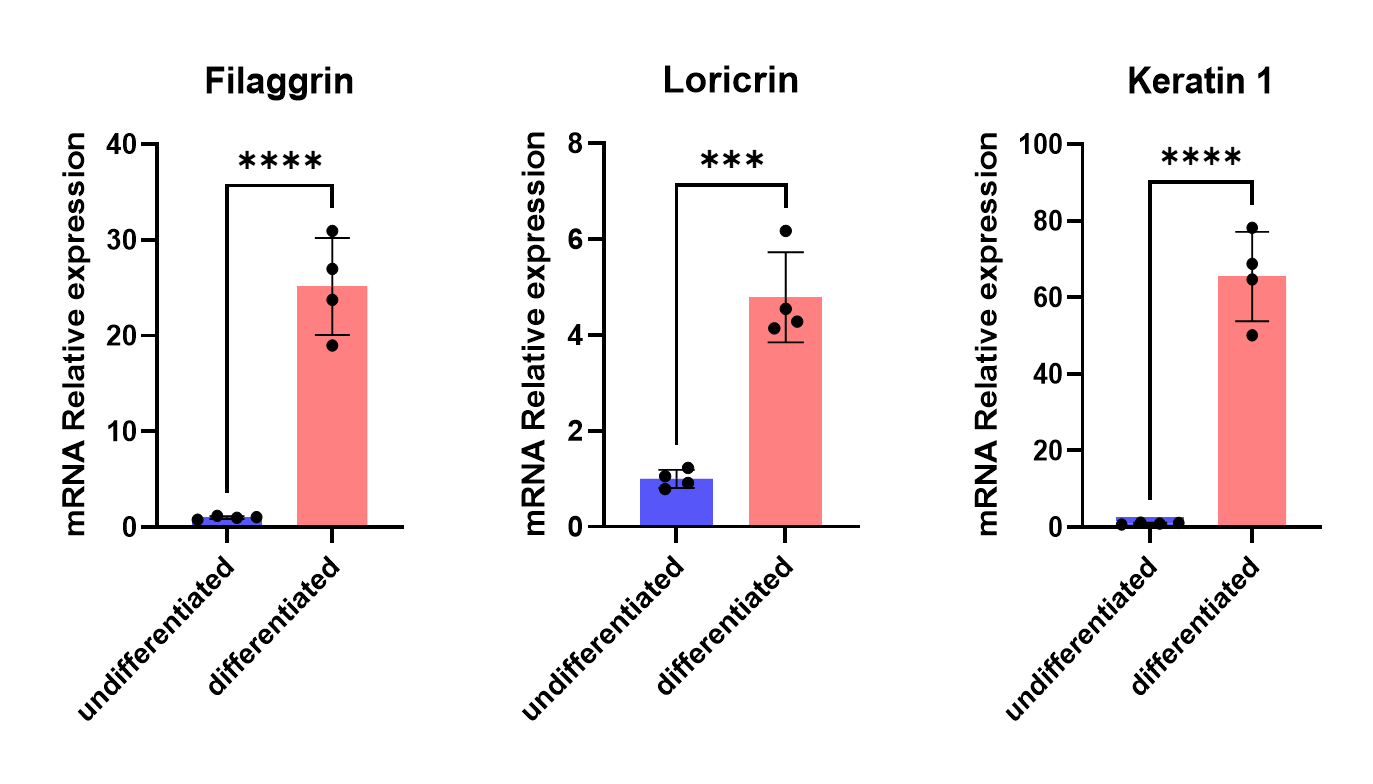


**Supplementary Figure 8. RT-qPCR gene expression analysis of keratinocytes.**

The mRNA levels of filaggrin, loricrin and keratin 1. RPLP0 was used as an internal control. Bars and lines represent mean ± SD. ***: p < 0.0002, ****: p < 0.0001.
